# Impact of family physician integrated care program on decreasing utilization of emergency department visit and hospital admission: a population-based retrospective cohort study

**DOI:** 10.1186/s12913-020-05347-7

**Published:** 2020-05-26

**Authors:** Po-Tsung Huang, Pei-Tseng Kung, Wen-Yin Kuo, Wen-Chen Tsai

**Affiliations:** 1 Department of Family Medicine, China Medical University Hospital, Taichung, Taiwan; 2 Department of Healthcare Administration, Asia University, Taichung, Taiwan; 3 Department of Medical Research, China Medical University Hospital, China Medical University, Taichung, Taiwan; 4 Department of Health Services Administration, China Medical University, 91, Hsueh-Shih Road, Taichung, Taiwan 40402

**Keywords:** Family physician integrated care program (FPIC), Emergency visits, Hospital admissions, Family physician

## Abstract

**Background:**

Hospital admission and emergency department(ED) visits are a massive burden in medical expenditures. In 2003, the Taiwanese government developed Family Physician Integrated Care Program (FPIC) to increase the quality of primary care and decrease medical expenditures. This study’s goals were to determine whether FPIC decreased hospital admissions and ED visits and identify the factors influencing the outcomes.

**Methods:**

This nationwide retrospective cohort study was conducted for the period between 2006 and 2013 by using data obtained from the Taiwan National Health Insurance Research Database. A total of 68,218 individuals were divided into those who joined FPIC and those who did not. We used propensity score matching at a ratio of 1:1 and logistic regression with the generalized estimating equation (GEE) model having a difference-in-difference design to investigate the effects of the FPIC policy on hospital admissions and ED visits in 7 years.

**Results:**

Using logistic regression with the GEE model with the difference-in-difference design, we found no reduction in ED visits and hospital admissions between the two groups. The participants’ risk of hospital admissions increased in the first year after joining FPIC (OR: 1.11, 95% CI: 1.03–1.20, *P* < .05). However, participants who joined FPIC showed an 8% lower risk of hospital admissions in the sixth and seventh years after joining FPIC, compared with those who did not join FPIC (OR: 0.92, 95% CI: 0.85–1.00, *P* < .05).

**Conclusions:**

FPIC in Taiwan could not decrease medical utilization initially but might reduce hospital admissions in the long term.

## Background

Emergency department (ED) utilization plays a vital role in health care expenditure. ED visits have been increasing over the past decades. In 2011, more than 400 ED visits for every 1000 individuals were reported in the population in the United States, indicating the lack of and poor access to community-based primary care [1]. Approximately 5.5–46.0% of ED utilization could be replaced by alternative medical facilities, which could save approximately $4.4 billion annually [2]. In Taiwan, the ED utilization rate for each person annually was reported to be approximately 18.1%, in which 15 and 20% of ED visits were nonurgent and preventable, respectively, as well [3]. Strategies should be developed to reduce ED utilization and medical expenditures.

Research has shown that higher quality and continuity of primary care may decrease ED utilization related to diabetes mellitus, congestive heart failure, dementia, and asthma [4–7]. Studies have reported that case management for high-risk patients may decrease ED utilization [8, 9]. Patient-centered medical homes program can lower overall ED utilization [10]. Lack of regular primary care and difficulties in accessing primary health care may lead to inappropriate ED utilization [11].

Hospital admissions due to poor control of chronic diseases account for a large portion of medical expenditures. In 2004, the costs of hospital admissions associated with short-term complications of diabetes or poor glycemic control in the United States exceeded US$1.3 billion [12]. High quality of primary care in diabetes could also reduce the chance of hospital admissions [5, 13]. Research indicated that the rate of hospital admissions induced by poor control of chronic diseases could serve as one of the significant indicators of primary care quality [14]. Consequently, improvement of the quality of primary care and reduction of hospital admissions could be significantly related.

The World Health Organization (WHO) indicated that primary health care should be emphasized by implementing community care networks that provide patient-centered care [15]. Many similar care models have been implemented in several countries, including Patient-Centered Medical Homes and Accountable Care Organizations in the United States, Multidisciplinary Medical Homes in France, Primary Care Federations in the United Kingdom, a team-based primary care model in Canada, and Family Physician Integrated Care program (FPIC) in Taiwan [10, 16, 17].

In 2003, the Taiwanese government developed FPIC to improve primary health care quality by implementing case management, establishing a referral system, providing 24-h phone consultation, and increasing the preventive health screening rate. In FPIC, 5–10 primary care physicians established a community care group, and some patients voluntarily became members of the community care group. A government authority could assign other patients with high medical expenditures in the previous year to each group. The annual management fee per patient member was 250 NTD (New Taiwan Dollar), which is approximately US$8. The quality indicator achievement fee was 550 NTD (approximately US$18) per member per year. Quality indicators included members’ ED visits and hospital admission rates, members’ satisfaction, and preventive health screening achievement rates. The quality achievement fee would be paid according to the proportion of indicators fulfilled [18].

The most similar policy to FPIC is the team-based primary care model in Canada [19]. Research indicated that the team-based primary care model could decrease primary care visits per patient per year by 11% and could decrease the total expenditure for specialist visits by 6%. However, the model could not decrease ED utilization and hospital admission rates [20]. Besides, a systematic review indicated that the team-based primary care model could reduce members’ ED visit rates, but mixed results were obtained for hospital admissions [21]. Few studies have been conducted on FPIC in Taiwan; hence, evidence regarding the outcome of FPIC is not available. Accordingly, the goals of this study were to determine whether FPIC decreased ED utilization and hospital admission rates and identify the factors influencing FPIC.

## Methods

### Study design

This retrospective longitudinal cohort study analyzed data obtained from a secondary database, namely the Taiwan National Health Insurance Research Database (NHIRD), for the period between 2006 and 2013; this database is maintained by the National Health Insurance Administration of Taiwan. The database includes the medical records of all individuals insured by the National Health Insurance program in Taiwan. In 2016, a total of 23,719,229 people were insured, constituting 99.60% of Taiwan’s population. In this study, we used three subsets with a total of 2,651,348 individuals randomly sampled from the NHIRD. No statistical differences were observed in the distribution of gender, age, or premium-based monthly income between individuals in the subsets and those in the original NHIRD [22].

### Participants

A total of 2,651,348 participants were selected from the three subsets of the NHIRD in 2006. We recruited participants who joined FPIC only in 2006. Participants who were less than 18 years old, died during 2006–2013, and had missing data were excluded. Finally, this study included 1,635,524 participants, including 34,109 participants who joined FPIC (FPIC group). Participants and non-participants were matched with a ratio of 1:1 based on the propensity score with a greedy matching technique according to the control variables, including gender, age, monthly income, urbanization level of residence areas, and Charlson comorbidity index (CCI) [23]. After matching, we identified 34,109 participants as the control group. We followed up the two matched groups from 2006 to 2013 to identify ED visits and hospital admissions.

### Measurements

The study participants were categorized by gender (male and female), age (divided into five groups from < 45 y to ≥75 y), monthly income (divided into seven groups), urbanization level of residential area (on a scale of 1 to 7, [24] with 1 indicating the most urban area), CCI score (divided into four groups from 0 to ≥3 points), the annual frequency of outpatient visits (divided into six groups from 0 to 5 to ≥25 visits), level of medical institutions (medical center, regional hospital, local hospital, and clinic), and ownership of medical institutions (public or nonpublic).

### Statistical analysis

The chi-square test was used to examine the difference in categorical data, including gender, age, monthly income, urbanization level of the residential area, CCI, the annual frequency of outpatient visits, level of medical institutions, and ownership of medical institutions. To investigate how FPIC influenced ED visits and hospital admissions, we applied logistic regression with the generalized estimating equation (GEE) model. The study had a difference-in-difference design, with adjustments for gender, age, monthly income, urbanization level of residential areas, CCI, the annual frequency of outpatient visits, level of medical institutions, and ownership of medical institutions. For all analyses, a *P*-value of <.05 was considered statistically significant. All statistical analyses were conducted using SAS software (Version 9.4, SAS Institute Inc., Cary, NC, USA). The Institutional Review Board of China Medical University approved this study (IRB No.: CMUH107-REC3–053).

## Results

As shown in Table 1, the characteristics, including gender, age, monthly income, urbanization level of residential areas, and CCI, of the FPIC group were matched to those of the control group. After propensity score matching, no statistically significant differences in essential characteristics were observed between the two groups (*P* > .05). The study population comprised more female participants (63.1%) than male participants (36.9%), and 56.23% were aged less than 45 years. Moreover, 51.40% of the participants had a monthly income between NT$17,281 (US$576) and NT$22,800 (US$760). We noted that 27.89 and 31.93% of the participants lived in residential areas with urbanization levels of 1 and 2, respectively. Furthermore, 77.79% of the participants did not have any comorbidity (CCI = 0).

**Table 1:** Multivariable analysis of patients who joined or did not join FPIC before and after matching

Variable	Before matching					After 1:1 matching				
	Control		FPIC		*P*-value	Control		FPIC		*P*-value
	N	%	N	%		N	%	N	%	
**Gender**					< 0.001					1.000
Female	808,221	50.47	21,523	63.10		21,523	63.10	21,523	63.10	
Male	793,194	49.53	12,586	36.90		12,586	36.90	12,586	36.90	
**Age**					< 0.001					1.000
< 45	987,867	61.69	19,179	56.23		19,178	56.23	19,179	56.23	
45–54	318,795	19.91	6811	19.97		6812	19.97	6811	19.97	
55–64	159,404	9.95	3910	11.46		3910	11.46	3910	11.46	
65–74	93,682	5.85	3082	9.04		3082	9.04	3082	9.04	
≥ 75	41,677	2.60	1127	3.30		1127	3.30	1127	3.30	
**Monthly salary (NTD)**					< 0.001					1.000
≤ 17,280	142,846	8.92	3154	9.25		3300	9.67	3154	9.25	
17,281-22,800	785,014	49.02	17,533	51.40		17,242	50.55	17,533	51.40	
22,801-28,800	171,992	10.74	3606	10.57		3395	9.95	3606	10.57	
28,801-36,300	143,006	8.93	2860	8.38		3092	9.07	2860	8.38	
36,301-45,800	164,786	10.29	3309	9.70		3276	9.60	3309	9.70	
45,801-57,800	85,035	5.31	1714	5.03		1715	5.03	1714	5.03	
≥ 57,801	108,736	6.79	1933	5.67		2089	6.12	1933	5.67	
**Urbanization level of residential area**					< 0.001					1.000
1	529,005	33.03	9514	27.89		9514	27.89	9514	27.89	
2	491,320	30.68	10,890	31.93		10,890	31.93	10,890	31.93	
3	256,205	16.00	6332	18.56		6332	18.56	6332	18.56	
4	198,003	12.36	4742	13.90		4743	13.91	4742	13.90	
5	25,627	1.60	612	1.79		611	1.79	612	1.79	
6	51,659	3.23	1174	3.44		1174	3.44	1174	3.44	
7	49,596	3.10	845	2.48		845	2.48	845	2.48	
**CCI**					< 0.001					1.000
0	1,365,137	85.25	26,534	77.79		26,534	77.79	26,534	77.79	
1	157,794	9.85	5000	14.66		5002	14.66	5000	14.66	
2	53,830	3.36	1784	5.23		1782	5.22	1784	5.23	
≥ 3	24,654	1.54	791	2.32		791	2.32	791	2.32	

**Fig. 1:**
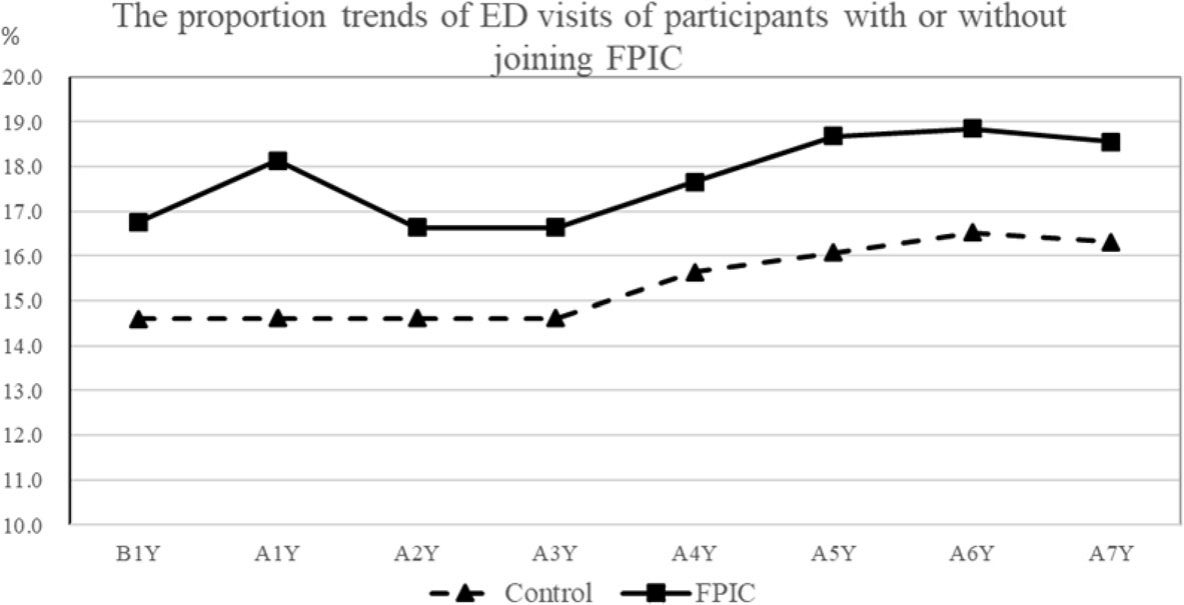
Proportion trends of ED visits of participants with or without joining FPIC. B1Y = 1 year before joining FPIC, AnY = nth year after joining FPIC

**Fig. 2:**
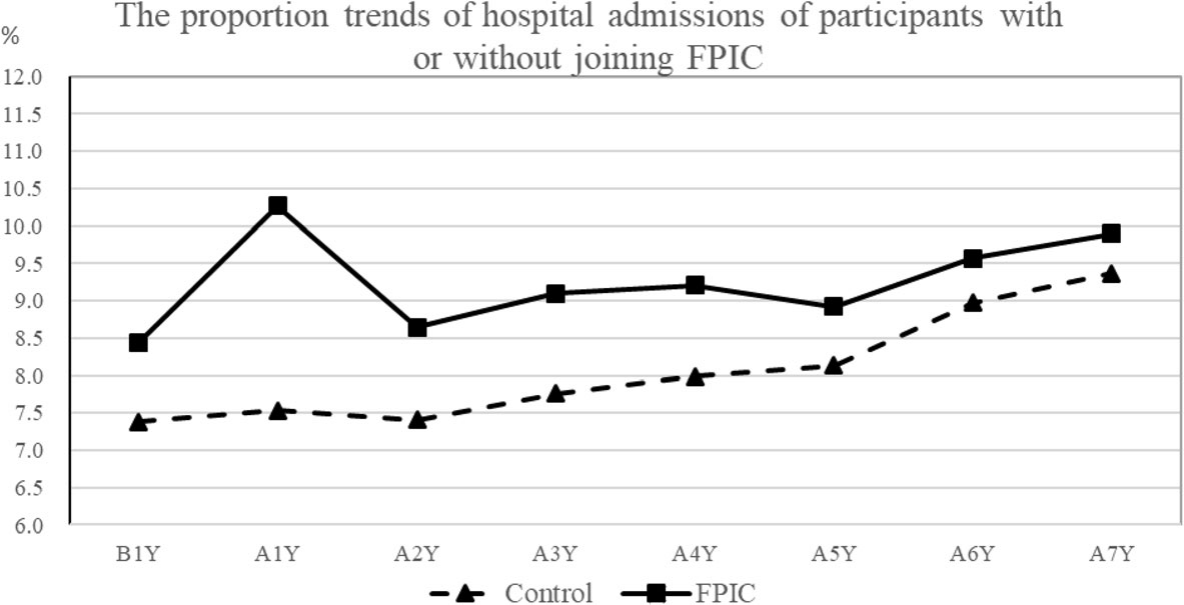
Proportion trends of hospital admissions of participants with or without joining FPIC. B1Y = 1 year before joining FPIC, AnY = nth year after joining FPIC

**Fig. 3:**
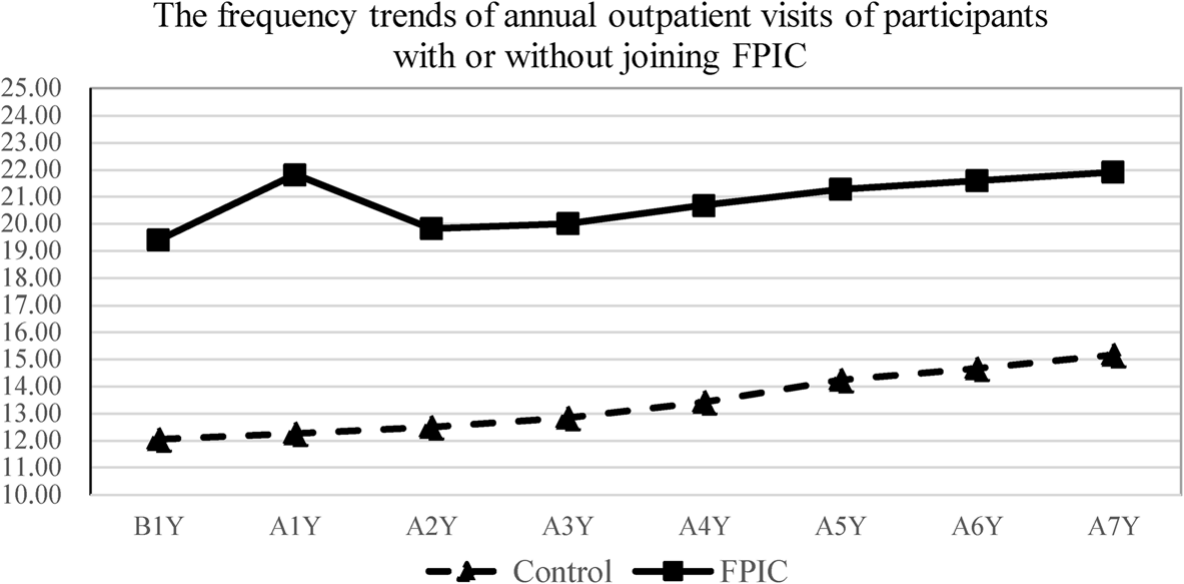
Frequency trends of annual outpatient visits of participants with or without joining FPIC. B1Y = 1 year before joining FPIC, AnY = nth year after joining FPIC

The proportion trends of ED visits and hospital admissions of participants who joined or did not join FPIC from 2006 to 2013 are shown in Figs. 1, 2. The frequency trends of annual outpatient visits are shown in Fig. 3. ED visits, hospital admissions, and outpatient visits in the FPIC and control groups gradually increased in the long term. The FPIC group had a higher proportion of ED visits, hospital admissions, and a higher frequency of outpatient visits than did the control group. Moreover, in the FPIC group, the participants’ ED visits, hospital admissions, and outpatient visits all increased in the first year after joining FPIC. They returned to baseline in the second year after joining FPIC.

We conducted logistic regression with the GEE model with the difference-in-difference design to determine whether FPIC influenced ED visits and hospital admissions after adjusting for the participants’ basic characteristics, health status, and hospital attributes; the regression results are presented in Table 2. No significant difference in ED visits was observed between the FPIC and control groups (OR: 1.01; 95% CI 0.03–0.06; *P* = .526). Compared with the participants’ ED visits in the year before joining FPIC, no significant difference was observed in their ED visits in every year after joining FPIC (*P* = .345–.762). Men were likely to visit the ED than women (OR: 1.15; 95% CI: 0.1–0.16; *P* < .001). Participants aged 45–74 years had fewer ED visits than did other participants (OR: 0.68–0.75, *P* < .001). Participants with higher monthly income tended to have fewer ED visits (OR: 0.89–0.74; *P* < .001). Also, participants who lived in a residential area with an urbanization level of 1 (the most urban area) had the lowest ED visits, without a consistent trend. Participants with higher CCI scores had higher ED utilization rates (OR: 1.11–1.83; *P* < .001). A higher frequency of annual outpatient visits was associated with higher ED utilization rates (OR: 1.35–3.25; P < .001). Participants who were mainly treated in clinics had the lowest ED visits (OR: 0.43; 95% CI: 0.42–0.45; P < .001) compared with those who were mainly treated in medical centers.

**Table 2:** Variables influencing ED visits and hospital admissions analyzed through logistic regression with the GEE model having a difference-in-difference design

Variables	ED visits				Hospital admissions			
	OR	95% C.I.		*P*-value	OR	95% C.I.		*P*-value
**FPIC or control**								
control (ref)								
FPIC	1.01	0.97-	1.06	0.526	0.99	0.93-	1.05	0.625
**Joining time**								
Before FPIC (ref)								
A1Y	1.01	0.97-	1.05	0.555	1.02	0.96-	1.08	0.467
A2Y	1.01	0.97-	1.05	0.767	0.99	0.93-	1.05	0.623
A3Y	1.00	0.96-	1.04	0.988	1.00	0.94-	1.06	0.895
A4Y	1.05	1.00-	1.09	0.029	0.99	0.93-	1.05	0.671
A5Y	1.06	1.02-	1.11	0.003	0.96	0.90-	1.02	0.156
A6Y	1.08	1.03-	1.12	< 0.001	1.03	0.97-	1.09	0.295
A7Y	1.03	0.99-	1.08	0.121	1.03	0.97-	1.09	0.312
**Policy effect**								
FPIC*A1Y	1.03	0.97-	1.08	0.359	1.11	1.03-	1.20	0.007
FPIC*A2Y	0.97	0.92-	1.03	0.348	1.00	0.92-	1.08	0.920
FPIC*A3Y	1.01	0.95-	1.07	0.762	1.02	0.95-	1.11	0.559
FPIC*A4Y	0.99	0.93-	1.04	0.664	1.01	0.93-	1.09	0.861
FPIC*A5Y	1.03	0.97-	1.09	0.345	0.96	0.88-	1.04	0.273
FPIC*A6Y	1.01	0.96-	1.07	0.640	0.92	0.85-	1.00	0.050
FPIC*A7Y	1.02	0.96-	1.08	0.551	0.92	0.85-	1.00	0.045
**Gender**								
Female (ref)								
Male	1.15	1.12-	1.17	< 0.001	1.01	0.98-	1.03	0.708
**Age**								
< 45 (ref)								
45–54	0.75	0.73-	0.77	< 0.001	0.59	0.57-	0.61	< 0.001
55–64	0.68	0.66-	0.70	< 0.001	0.57	0.55-	0.59	< 0.001
65–74	0.72	0.70-	0.75	< 0.001	0.68	0.65-	0.71	< 0.001
≥ 75	0.99	0.95-	1.03	0.728	0.87	0.83-	0.91	< 0.001
**Monthly salary**								
≤ 17,280 (ref)								
17,281-22,800	0.94	0.91-	0.98	0.001	1.03	0.98-	1.08	0.264
22,801-28,800	0.94	0.91-	0.98	0.003	0.99	0.94-	1.04	0.681
28,801-36,300	0.89	0.85-	0.92	< 0.001	0.97	0.92-	1.03	0.294
36,301-45,800	0.84	0.80-	0.87	< 0.001	0.94	0.89-	1.00	0.047
45,801-57,800	0.75	0.71-	0.79	< 0.001	0.84	0.78-	0.90	< 0.001
≥ 57,801	0.74	0.70-	0.78	< 0.001	0.76	0.71-	0.82	< 0.001
**Urbanization**								
1(ref)								
2	1.05	1.02-	1.07	< 0.001	1.09	1.05-	1.12	< 0.001
3	1.02	0.99-	1.05	0.114	1.16	1.11-	1.20	< 0.001
4	1.12	1.08-	1.15	< 0.001	1.27	1.22-	1.32	< 0.001
5	1.05	0.97-	1.13	0.220	1.18	1.08-	1.29	< 0.001
6	1.18	1.12-	1.25	< 0.001	1.45	1.36-	1.54	< 0.001
7	1.04	0.98-	1.11	0.174	1.29	1.19-	1.39	< 0.001
**CCI**								
0 (ref)								
1	1.11	1.09-	1.14	< 0.001	1.27	1.23-	1.31	< 0.001
2	1.24	1.20-	1.28	< 0.001	1.88	1.80-	1.96	< 0.001
≥ 3	1.83	1.75-	1.92	< 0.001	3.96	3.76-	4.17	< 0.001
**Frequency of annual outpatient visits**								
0 ~ 5 (ref)								
6 ~ 10	1.35	1.32-	1.39	< 0.001	2.34	2.20-	2.49	< 0.001
11 ~ 15	1.68	1.63-	1.73	< 0.001	4.01	3.77-	4.26	< 0.001
16 ~ 20	2.03	1.97-	2.10	< 0.001	6.02	5.66-	6.41	< 0.001
20 ~ 25	2.39	2.31-	2.47	< 0.001	8.03	7.54-	8.56	< 0.001
≥ 25	3.25	3.15-	3.35	< 0.001	10.85	10.22-	11.53	< 0.001
**Level of medical institutions**								
Medical center(ref)								
Regional hospital	1.13	1.09-	1.17	< 0.001	0.99	0.95-	1.03	0.738
Local hospital	0.91	0.88-	0.94	< 0.001	0.71	0.67-	0.74	< 0.001
Clinic	0.43	0.42-	0.45	< 0.001	0.23	0.22-	0.23	< 0.001
**Ownership of medical institutions**								
Public hospital (ref)								
Private hospital	1.02	0.99-	1.05	0.313	1.10	1.06-	1.14	< 0.001

AnY = nth year after joining FPIC

The relationship between FPIC and hospital admissions is shown in Table 2. We still observed no significant difference in hospital admissions between the FPIC and control groups (OR: 0.99; 95% CI: 0.93–1.05; *P* = .625). The participants’ risk of hospital admissions increased in the first year after joining FPIC (OR: 1.11, 95% CI: 1.03–1.20, *P* < .05). However, the participants’ hospital admissions decreased by 8% in the sixth and seventh years after joining FPIC, respectively (OR: 0.92; 95% CI: 0.85–1.00; *P* = .050; OR: 0.92; 95% CI: 0.85–1.00; *P* = .450). Compared with younger participants (aged less than 45 y), older participants had fewer hospital admissions (OR: 0.59–0.87; *P* < .001). Participants within the two highest monthly income categories had fewer hospital admissions (OR: 0.84–0.79; P < .001). Higher CCI led to more hospital admissions (OR: 1.27–3.96; *P* < .001). Participants who visited the outpatient department over 25 times per year had approximately 10 times more hospital admissions (OR: 10.85; 95% CI: 10.22–11.53; *P* < .001). Regarding the level of medical institutions, hospital admissions of participants treated mainly in clinics were 77% lower than those of participants treated in medical centers (OR: 0.23; 95% CI: 0.22–0.23; P < .001). Hospital admissions of participants treated in private hospitals were 10% higher than those of participants treated in public hospitals (OR: 1.10; 95% CI: 1.05–1.14; P < .001).

## Discussion

Our results reveal that the FPIC group showed no significant decrease in ED visits and hospital admissions compared to the control group within seven years. However, the FPIC group exhibited fewer hospital admissions in the sixth and seventh years after joining FPIC. It suggests that the FPIC policy implemented in Taiwan might have improved primary care quality after six years (long term) but had no significant effect in the short term. The primary care system in Canada, which integrates interdisciplinary practice with blended payment arrangements (i.e., combining fee-for-service with capitation or incentive payments), is very similar to Taiwan’s FPIC [17]. A previous study in Canada showed that family medicine groups (FMGs) had no effect on ED visits and hospital admissions but significantly reduced outpatient visits, suggesting an improvement of primary care quality [20], which is consistent with our study finding. However, a systematic review in Canada indicated that the primary care team-based model could reduce ED visits, but no consistent evidence was found for hospital admissions [21]. Mortality or morbidities engendered by chronic diseases may occur over 5–10 years, which may delay the policy’s effect. Our finding of no reduction of ED visits might be because no compulsory hierarchy has been established for medical care in Taiwan, where patients can directly visit any medical institution, including a medical center, without a referral.

Clinic doctors recruited the FPIC group, and a government authority could assign participants with higher medical expenditures in the previous year to the FPIC group; the higher medical expenditures indicated that these participants had higher medical utilization and more inferior health status. Therefore, as shown in Figs. 1, 2, and 3, the FPIC group had approximately 3% higher ED visits and hospital admissions and about seven more annual outpatient visits than did the control group before joining FPIC. Moreover, the FPIC group registered increased ED visits, hospital admissions, and annual outpatient visits in the first year after joining FPIC. This phenomenon might be related to the establishment of 24-h phone consultation centers in the FPIC policy. Such a 24-h consultation center was often set up in the ED of a medical center. It was challenging to identify the severity of the disease by medical practitioners who received emergency phone calls. Therefore, asking patients to visit the ED would be a reasonable suggestion, which led to increases in ED visits and hospital admissions. Furthermore, primary care physicians would suggest the patients of FPIC group to receive health exams for better care quality. Hidden diseases might be detected, which contributed to more outpatient visits.

In this study, male patients had higher hospital admissions, which is consistent with previous results [25, 26]. We also observed that younger patients had higher ED visits and hospital admissions, which is inconsistent with previous results [27, 28]. This finding might be related to higher trauma risk among younger patients and low medical costs in Taiwan. Previous studies have shown that patients with higher socioeconomic status have fewer hospital admissions [29–32] and ED visits [27, 33, 34]. Our study revealed consistent results; that is, patients who had higher monthly income and who were living in more urbanized residence areas had a lower possibility of ED visits and hospital admissions. A previous study showed that more frequent primary care outpatient visits were associated with higher hospital admissions, indicating that poor health condition is related to hospital admission [35]. A high annual frequency of hospital admissions and ED visits and a high CCI score implies patients’ poor health conditions. We observed consistent results that patients with more inferior health status had higher ED visits and hospital admissions.

In this study, patients mainly treated in local hospitals and clinics had fewer ED visits and hospital admissions than did those treated in medical centers. Receiving care mainly in clinics implied more favorable health conditions and more favorable primary care continuity and accessibility. This result is consistent with previous results that more favorable continuity and accessibility of primary care led to fewer ED visits [36, 37] and hospital admissions [5, 14, 38].

### Limitations

As a secondary database, the NHIRD has some limitations. These include the lack of data on individual health conditions and behaviors, such as body mass index, tobacco smoking, alcohol drinking, or exercise habits. Monthly income cannot wholly reflect socioeconomic status. Moreover, CCI can only reflect the significant part but not all of the health conditions. Although we have already used frequency of annual outpatient visits as covariate adjustment in logistic regression for more valid results, we did not match patients with similar utilization in the year before joining FPIC, which is our study limitation.

## Conclusion

Our study revealed that FPIC could reduce hospital admissions in the long term but did not affect ED visits. According to our results, policymakers should adjust FPIC to achieve more robust outcomes. Moreover, further research should examine the other effects of FPIC, such as those on cost-effectiveness, outpatient health care utilization, or health outcomes.

## Data Availability

Data are available from the Health and Welfare Data Science Center of the Ministry of Health and Welfare (MOHW) (http://www.mohw.gov.tw/EN/Ministry/Index.aspx), Taiwan. All interested researchers can apply for using the database managed by the MOHW. Due to legal restrictions imposed by the Taiwanese government related to the Personal Information Protection Act, the database cannot be made publicly available. Any raw data are not allowed to be brought out from the Health and Welfare Data Science Center. The restrictions prohibited the authors from making the minimal data set publicly available.

## Abbreviations

ED: Emergency department
FPIC: Family Physician Integrated Care Program
GEE: Generalized estimating eq.
OR: Odds ratio
CI: Confidence interval
CCI: Charlson comorbidity index
WHO: World Health Organization
NHIRD: National Health Insurance Research Database
NTD: New Taiwan Dollar
B1Y: 1 year before joining FPIC
AnY: Nth year after joining FPIC
Ref: Reference group
